# Early warning signals from the periphery: A model suggestion for the study of critical transitions

**DOI:** 10.1007/s42001-021-00142-8

**Published:** 2021-09-15

**Authors:** Manfred Füllsack, Daniel Reisinger, Marie Kapeller, Georg Jäger

**Affiliations:** Institute of Systems Sciences, Innovation and Sustainability Research at the University of Graz, Graz, Austria

**Keywords:** Critical transitions, Early warning signals, Social interaction, Agent-based modeling, Equation-based modeling, Hysteresis

## Abstract

Studies on the possibility of predicting critical transitions with statistical methods known as early warning signals (EWS) are often conducted on data generated with equation-based models (EBMs). These models base on difference or differential equations, which aggregate a system’s components in a mathematical term and therefore do not allow for a detailed analysis of interactions on micro-level. As an alternative, we suggest a simple, but highly flexible agent-based model (ABM), which, when applying EWS-analysis, gives reason to (a) consider social interaction, in particular negative feedback effects, as an essential trigger of critical transitions, and (b) to differentiate social interactions, for example in network representations, into a core and a periphery of agents and focus attention on the periphery. Results are tested against time series from a networked version of the Ising-model, which is often used as example for generating hysteretic critical transitions.

## Introduction

For anticipating regime shifts in dynamic systems a set of methods has been suggested, which is commonly referred to as early warning signals (EWS) [1, 2]. Particularly in ecology, EWS-analysis is often applied to state changes from accepted to detrimental conditions, which occur abruptly compared to the rather slow development of a critical parameter that is assumed to drive the shift [3–5]. Together with some stochasticity in the system, this makes the shifts difficult to predict. Therefore, they are commonly referred to as critical transitions.

Critical transitions are thought to arise from strong non-linear interactions in a system’s components, causing so-called bifurcations, that is, equilibrium changes in a system’s behavior from a stable to an unstable state or back. Particularly interesting are changes where a system’s state shifts to and fro at different values of a (slowly driven) control or “critical” parameter. Between these values, the system may remain in a bi-stable phase, where the point of transition, the tipping point, depends on the system’s history, that is, on the direction from which the critical parameter is driven. This phenomenon is known as hysteresis [6]. Climate change for example, as it is thought to be caused by high CO2-emissions, is suspected to be subject to such hysteresis, implying that once the regime shift to a warm climate has happened it cannot easily be reverted, because going back to a more moderate climate would necessitate a reduction of much more CO2 than originally caused the warming [7]. Similar has been observed in economic [8] and social systems [9].

The subtleties of critical transitions raise demand for fine-tuning EWS-analysis to further fathom its potential. In this regard, it seems useful to have a detailed understanding of the drivers of critical transitions. One way to obtain this is through computer-based simulations, with EWS-investigations to a large extent focusing on generating data with equation-based simulation models (EBM). As will be shown, however, this method tends to veil certain aspects of the causes of critical transitions. For this reason, this paper undertakes to dig into the details of these causes with the help of an agent-based model loosely oriented on spin models of ferro-magnetism, that was created in order to separate what could be called a “factual” influence of a critical parameter from the “social” influence of the system’s components interaction. We believe that this separation suggests some reconsiderations of the application of Early Warning Signals in attempts to anticipate critical transitions.

The paper is organized as follows: the next section will discuss some deficits of representing critical transitions with EBMs and models like the Ising model of ferro-magnetism. The following section will introduce the suggested ABM and discuss its implications. The next section will apply EWS to time series generated with the grid-version of this model. The following section will expand the EWS analysis to networked versions of the model. The next section will discuss the findings from the model on the background of a similar analysis of a networked version of the Ising-model and conclude the paper.

## Critical transitions

**Fig. 1:**
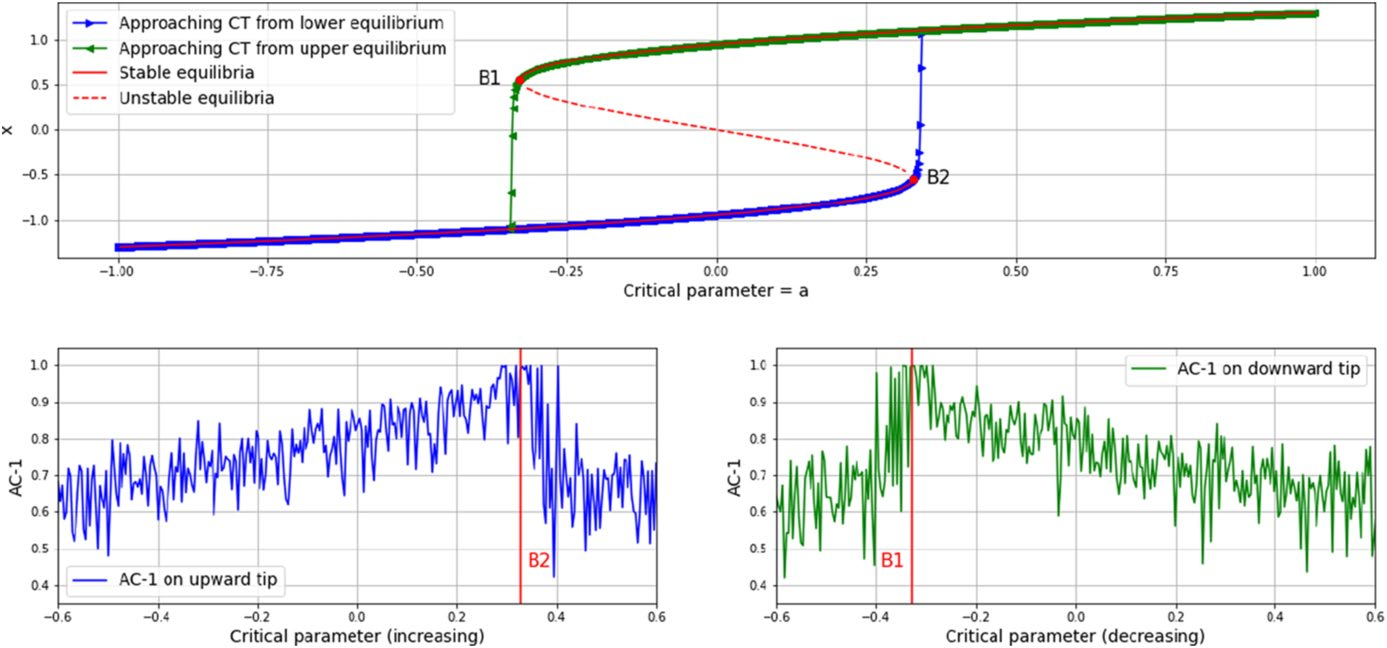
Numerical simulations of the system expressed in equation (1), with $c$ = 0.9. In order to indicate the potential of EWS-analysis (discussed in “EWS-analysis on the grid version”) on this example, the auto-correlation with lag-1 (AC-1) of the blue (bottom left, $a$ increasing) and green (bottom right, $a$ decreasing) curves in the top plot are shown. In both cases, AC-1 clearly increases towards 1 in approaching the tipping points (indicated with red vertical lines)

Rapid state changes are considered a consequence of a non-linear relationship of system components, suggesting a reinforcement dynamic, or in other words, a positive feedback effect of some development that gets stronger the more it is driven. An illustrative example is the occurrence of avalanches. Usually, this “rich-get-richer”-reinforcement is specified as *social* [10, 11], pointing at interactions and neighborhood or peer effects as the actual inducer of critical transitions [12–15]. Particularly, diffusion theories in the wake of [16] are stressing the role of positive feedbacks in abrupt spreads of innovations, rumors or viruses for instance [17, 18]. An interesting additional effect in these transitions is hysteresis, which refers to a delay of a regime shift in relation to a tipping point as it would be suggested by the control parameter that drives the system’s change [6]. Hysteresis causes a delay in the shift in dependence of whether the parameter increases or decreases, thus making the system sensible to its history and consequently difficult to predict. Most commonly, model suggestions for simulating such transitions build on differential equations. One of the simplest examples may be the following:

1 $$\begin{aligned} x'=-x^3+cx+a \end{aligned}$$

which among others has been suggested by [19] to model economies coupled by trade, or patches of ecosystems coupled by movement of organisms. The top plot in Fig. 1 shows a numerical simulation of the system’s variable *x* undergoing critical transitions in dependence of whether the parameter $a$ increases (blue curve) or decreases (green curve), with the parameter $c$ determining the distance between the transitions. The equilibria curves of the system are shown in red.

The bottom-row plots in Fig. 1 show one of the commonly applied EWS-metrics—auto-correlation with lag-1 (AC-1)—in the onset of the transitions, with the left one approaching the bifurcation point B2 from the lower equilibrium (from left), and the right one approaching the bifurcation point B1 from the upper equilibrium of the system (from right). As is clearly visible, AC-1 increases towards 1 in the onset of the transitions. As will be explained in “EWS-analysis on the grid version” in greater detail, rising AC-1 therefore is taken to indicate an imminent state transition.

Such systems and the time series of their state variables can be reproduced with mathematical models, but these models rarely allow for an in-depth analysis of sub-dynamics that interact in generating the observable system dynamics, such as the interaction of different particle clusters for instance. This seems particularly adverse in the context of social or ecological systems where modeling is deployed for investigating the potential of EWS-metrics to anticipate detrimental transitions [1, 4, 20–22].

In physics,—the discipline where the phenomenon of hysteresis was discovered in the magnetization of metals [23], the role of interactions in state changes is explicit. In magnetism for instance, explanations point at the so-called Barkhausen-effect, arising through interacting groups of particles, so-called Weiss-domains, in uniform materials which can move to respond to a change in an external field. Due to disruptions from domain boundaries or non-magnetic inclusions, these domains may interlock for short times when moving and then, when the change in the magnetic field becomes strong enough to outweigh the local energy barrier, make groups of atoms flip their spins together, causing magnetization to proceed not continuously, but in minute jumps (which can be made audible as Barkhausen-noise). The aggregation of these interlocks may delay the overall spin-orientation in relation to the influence of the external field, thus causing hysteresis.

The probably most famous model suggested to simulate this spin-shifting process, the Ising-model [24], catches some of these aspects (see below). However, in its common implementations, which are also the ones that are often used in non-physical contexts (see below), it tends to obfuscate the causes by either averaging the process in a rather coarse mean-field approximation, or by packing it into an exponential function which complicates a detailed analysis of involved dynamics.

In its 2D-version [25], the Ising model represents magnetic dipole moments of atomic spins as points on a lattice which can be in one of two states ($+1$ or $-1$) dependent on an ambient temperature $t$ and an external magnetic field $h$. Spins are seeking a low energy state causing them to flip abruptly depending on a potential gain in energy. Consequently, as temperature increases, flipping to a higher energy state becomes more likely. In addition, however, flipping also depends on the state of a particle’s neighbouring particles. This interaction can create clusters of equal spin alignments causing clusters to flip together, thus producing the tiny jumps that determine the progress towards overall spin alignment as described by Barkhausen. At certain parameter values, these neighborhood interactions can be observed as scale-free distribution of cluster sizes with equal spin.

When raising temperature $t$ in the model, spin transitions show in the form of a pitchfork-bifurcation [26]. But when considering the magnetic field $h$ as critical parameter, the system develops a bi-stable region with two stable and one unstable states, creating a combined saddle-node bifurcation and thus an instance of hysteresis. Particularly, this dynamic is also often considered in non-physical contexts to represent social, economic or ecological regime shifts, where spatial interactions and neighbourhood influence matter [27–31]. In these cases, the binary spin mode is taken as pro ($+1$) and con ($-1$) of an opinion for instance, or the buying or selling of stocks, or the rejection or adoption of innovations, among others.

**Fig. 2:**
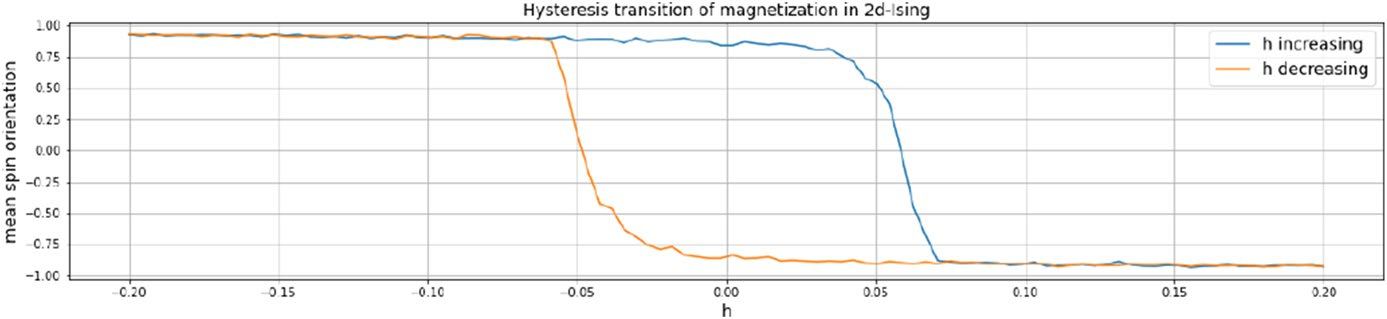
Mean aggregated magnetization in 2D-Ising model with a fixed temperature of $t = 2.12$ and an external magnetic field $h$ as critical parameter varied linearly between $-0.2$ and $0.2$, with magnetization undergoing phase transitions at different $h$-values depending on whether $h$ increases (blue) or decreases (yellow)

The model is commonly implemented in two versions: in an equation-based mode as a mean-field approximation of particle interaction, where the system’s sole observable is the aggregated magnetization. The second version is a sort of cellular automaton in a Von-Neumann neighborhood, which offers the possibility to consider particle interaction [25]. Usually, it is simulated in a Monte Carlo mode [32], deriving a probability for flipping the spin of a particle through taking the exponential of the ratio of temperature $t$ and negative energy that a particle can gain by flipping. The potential gain in energy is calculated as:

2 $$\begin{aligned} E_{diff} = 2s(nb-h)) \end{aligned}$$

where $s$ is the spin of the considered particle, $nb$ is the sum of the spins of the particle’s neighbors and $h$ is the external magnetic field, serving as the critical parameter in this case. The particle is then considered to flip its spin

3 $$\begin{aligned} if \quad E_{diff} \le 0\quad or\quad p < exp\left( \frac{-E_{diff}}{t}\right) \end{aligned}$$

where $t$ is the temperature and $p$ is a random real number between 0 and 1. The mean of the particles’ spins is taken as the observable aggregated magnetization. If the model is run with a fixed temperature of $t = 2.12$ and the external magnetic field as critical parameter varied linearly between $-0.2$ and $0.2$, magnetization undergoes a distinct phase transition, the actual tipping of which depends on whether $h$ increases or decreases (see Fig. 2).

**Fig. 3:**
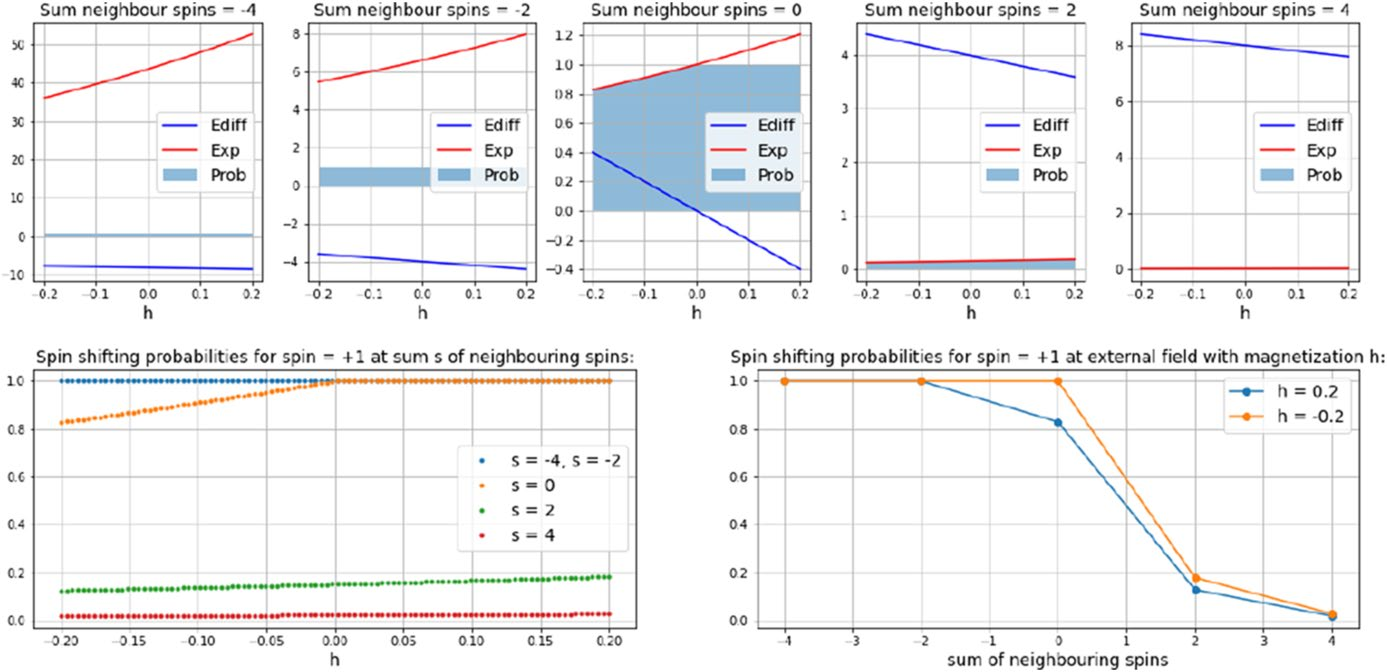
Neighborhood interaction in the dynamics of 2D-Ising model, assessed by considering equations 2 and condition 3 separately with regard to the sum of neighboring spins (top-row plots) and as spin shifting probabilities in dependence of the sum of neighboring spins with regard to the external field $h$ (bottom left) and in dependence of the considered extremes of $h$ ($-0.2, 0.2$) with regard to the sum of neighboring spins (bottom right)

The role of the neighborhood interaction in the Ising can be assessed to some extent when the potential energy gain $E_{diff}$ and the exponential function $exp(\frac{-E_{diff}}{t})$ are considered separately for each possible sum of neighbouring spins (top row plots in Fig. 3). As can be seen, a particle with spin = +1 in a neighborhood of either 3 or 4 particles with spin = +1 (spin sums 2 and 4, second right and right top row plots) faces a rather small probability to flip its spin ($p< 0.2$, resp. $p< 0.05$, the blue-grey area in the top row plots). The intensity of the external field $h$ has little influence (red and green lines in left bottom plot). A neighborhood with a tie of spins (and a spin sum of 0 therefore, middle top row plot) raises the probability of a spin flip. In the range between – 0.2 and 0 the critical parameter $h$ gains influence, but the difference to the positive $h$-values is small (about 18%). With 3 or 4 neighboring particles with a spin = -1 (second left and left top row plots), the first part of condition (3) takes effect, raising the flipping probability for the +1-particle constantly to 100%, irrespective of $h$. The values for the energy gain ($E_{diff}$ blue) and for the exponential function ($Exp$ red) lay far beyond the probability range and have no influence at all. For particles with spin $-1$, effects are exactly symmetric.

Taken together, the external field $h$ appears to have a surprisingly small range of impact on the overall transition dynamics, given the fact that it is the one parameter that changes (since $t$ is fixed at 2.12). Really noteworthy, the effect of $h$ is only with a neighborhood tie (spin sum = 0) and an $h$ in the range $-0.2< h < 0$. Its principle role in the hysteretic Ising, rather than to be the main driver in its own right, seems to be a sort of trigger for the positive feedback of neighborhood interaction. The actual transition dynamics are governed by the social influence. This might be a point which could be missed in the usual equation focused representation of the Ising-model.

## A model suggestion

**Fig. 4:**
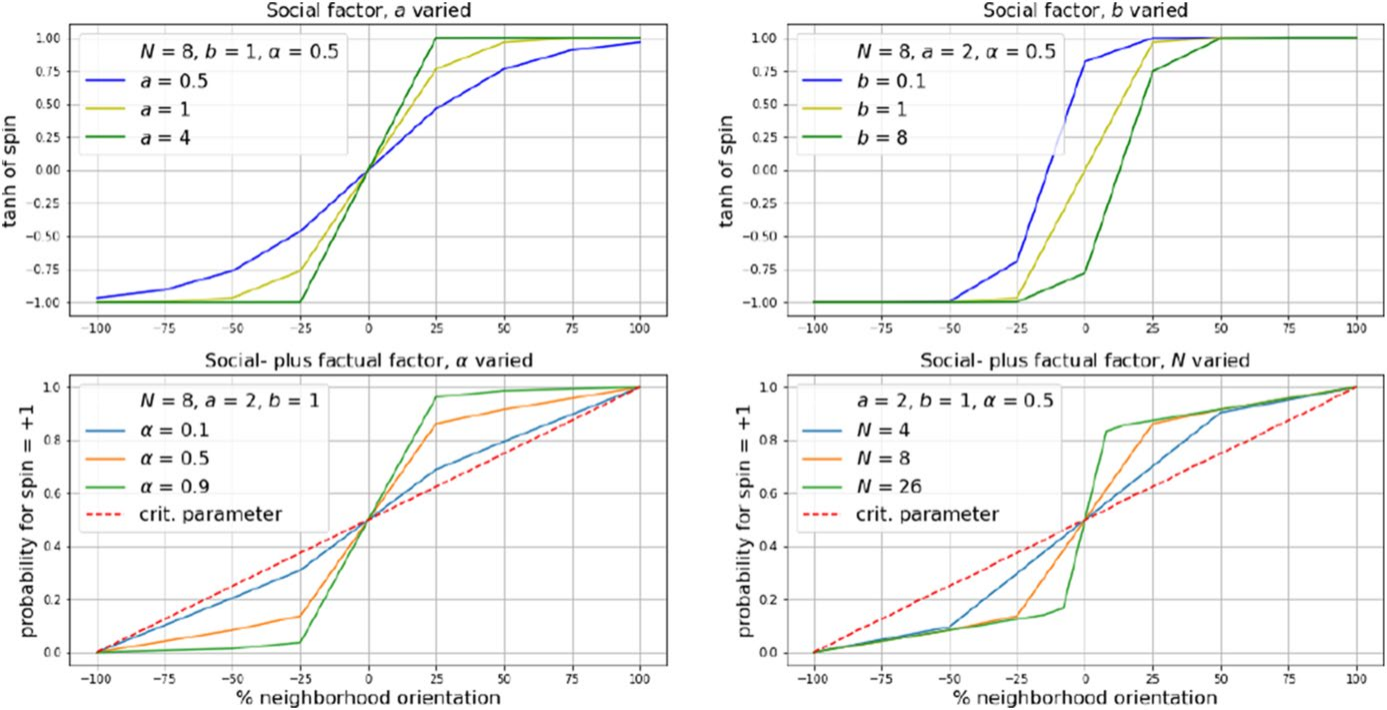
Results of varying parameters in regard to the percentile fraction of neighbors with spins either $-1$ or $+1$. Top-row plots showing the social factor on its own, bottom-row plots showing social plus factual factors scaled, varied in regard to the $\alpha$-weight for influence (bottom left plot), and varied with regard to $N$ the size of the neighborhood considered (bottom right plot)

For this reason, we undertook some comparative EWS-experiments with an alternative spin model that is, though differently based, inspired by the Ising-model and allows to separate what we call a “factual” and a “social” (or neighborhood) influence. A timely example to explain the rationale behind this might be the distinction of the “factual” effect of a Covid 19 vaccine from the “social” influence of rumours about its alleged side effects on the number of people who choose to be vaccinated. The model we suggest is simple to the extent that it defines the distribution of “social” influence beforehand through a function, which compresses the actual spin orientation into a relatively small interval around the tie of a particle’s neighbors with opposing orientations (see Fig. 4). This function reads:

4 $$\begin{aligned} f(x)=1-\frac{2b}{e^{ax}+b} \end{aligned}$$

and corresponds to the *tangens hyperbolicus* function when $a = 2$ and $b = 1$. With $a > 2$ ($a< 2$), it narrows (widens) the interval around the tie of neighbors and thus steepens (flattens) the transition. With $b > 1$, it shifts the midpoint of the transition to the right, with $0< b < 1$, it shifts it to the left, which could be interesting when social influence is thought to be asymmetrically distributed between conservative and progressive neighbors. $f(x)$ then simply is added to the one parameter that changes when the model is run, hence to the “critical” parameter $c$, which we consider as the “factual” influence, with both terms being weighted in regard to an $\alpha$-value representing the predominance of the one term over the other. The sum is normalized to the interval $0, 1$ and taken as a probability $p$ for a particle’s spin to be in state $+1$. In mathematical terms:

5 $$\begin{aligned} p=norm(\alpha f(s+nb) + (1-\alpha ) c) ,=> \{0,1\} \end{aligned}$$

where $s$ is the spin of the considered particle (or agent), $nb$ is the sum of the spins of its neighbors, $c$ is the critical parameter (the “factual” influence), $\alpha$ is the weight expressing the predominance of social or factual influence and $norm$ provides a Min-Max-normalization for the interval $0, 1$^1^. Figure 4 shows different results of varying these parameters in regard to the percentile fraction of neighbors with spins either $-1$ or $+1$, with the top row plots showing the social factor on its own and the bottom row plots showing social plus factual factors scaled, varied in regard to the $\alpha$-weight for influence (bottom left plot), and varied with regard to $N$ the size of the neighborhood considered (bottom right plot).

**Fig. 5:**
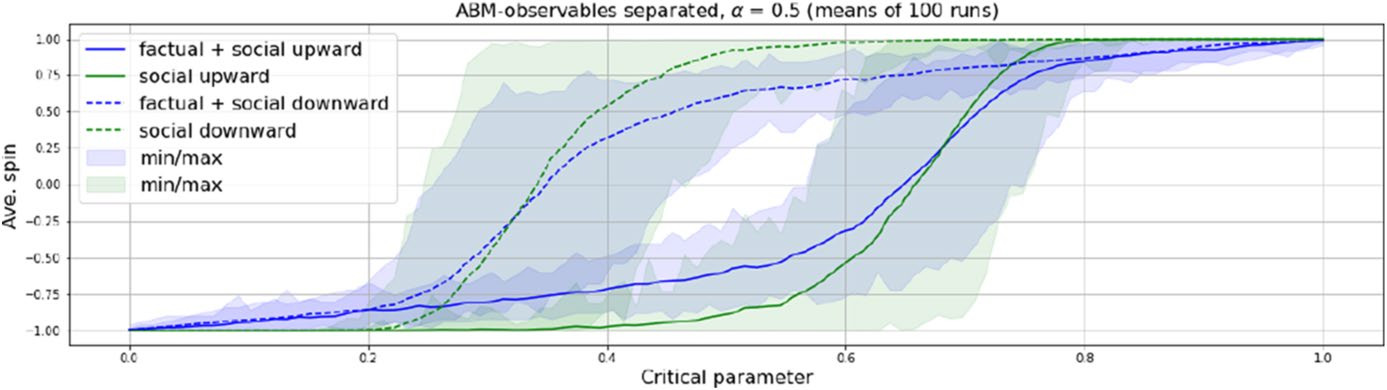
Observables from ABM with separated neighborhood-influence. Blue curves: average spin orientation of agents in grid-topology with Moore-neighborhood ($N=8$); green curves: neighborhood influence before being summed with critical parameter. ($\alpha =0.5$, i.e. equal influence of social and factual influence)

This analytical separation of social and factual influence allows to observe the reaction of the two terms separately in each step of time. Note that the intensity of social influence (i.e. the combined influence of neighbors with same spin) nevertheless is determined by the history of the system up to the point of observation. At this point however, it can be observed before it gets summed with the critical parameter to provide the next step’s spin orientation. This setting thus allows for a second aggregated observable, namely social influence by itself (green curves in Fig. 5), in addition to the average spin orientation (i.e. magnetization in the Ising, blue curves in Fig. 5).

In order to fully account for neighborhood interaction, and different from the Ising, the model is not allowed to converge over several time steps in order to equilibrate. Since $p$ serves as probability for a spin being in state $+1$, and not as a flipping threshold as in the Ising, equilibration here would mean to simply let the particles replicate the probability curve by way of Monte Carlo simulation (i.e. by the law of large numbers). If instead the system states are taken at each time step without convergence, the particles spontaneous interaction causes their spins to cluster and thus to delay the transition in relation to where it should occur according to probability. This seems to more closely replicate natural clustering processes (if they are not e.g. subjected to simulated annealing). The result is a typical hysteresis delay in regard to the upward and downward driven critical parameter, as shown in Fig. 5.

**Fig. 6:**
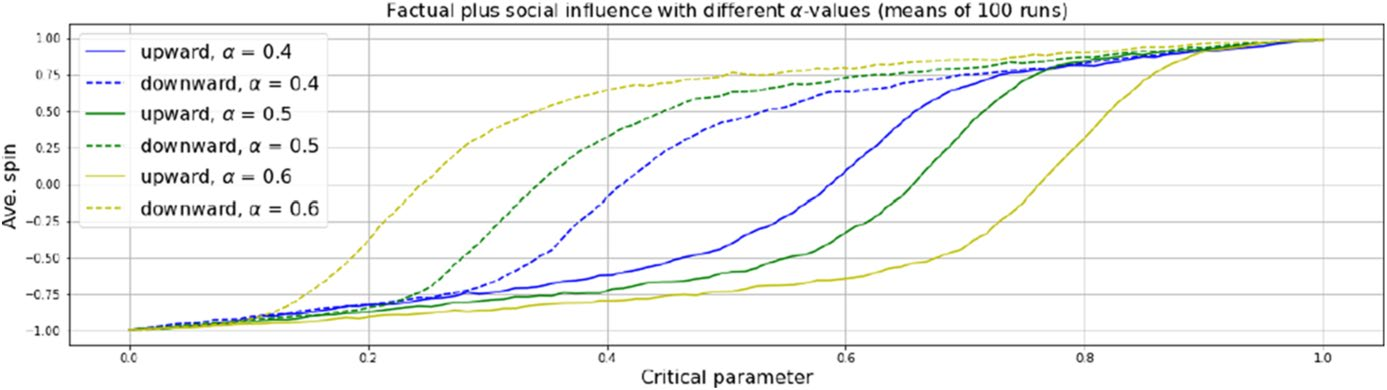
Spin-orientation transitions in ABM in relation to $\alpha$-weighted social and factual influence with upwards and downwards driven critical parameter. Rising predominance of social influence (increasing $\alpha$) increases the delay of the transitions

As can be expected, this delaying effect is amplified through giving additional weight to what here is called social influence. As mentioned, our model setting allows to consider a predominance of one kind of influence over the other, which could be particularly interesting when modeling transitions in social or economic contexts, where it is common to consider effects of rumor mongering or peer pressure. Various studies have shown that social effects can outweigh factuality and lead to stable believes beyond evidence [33, 34] or to speculation bubbles [35] or to rejections of innovations despite rational advantages [36], etc. With $\alpha =0.5$ in equation 2, the influence of neighbors and the one of the critical parameter, i.e. social and factual influence are taken to be equal. If $\alpha <0.5$ factuality prevails, if $\alpha >0.5$ social influence dominates. The weighting supports the thesis that it is social influence, i.e. particle interaction, which is the prime driver of hysteresis. The higher the predominance of social influence, the more the transitions get delayed (Fig. 6), a fact that might easily be missed in EBM-based representations of hysteresis systems.

## EWS-analysis on the grid version

The possibility to observe social influence separately as a central driver of critical transitions may provide new options for EWS-analysis. To show this, in a first step we compare the signals of the standard observable, the spin average, and the observable added here, the social influence by itself, and apply several commonly deployed EWS-metrics to them while varying the $\alpha$-parameter, which determines the weight given to factual and social influence.

Most EWS-metrics build on a phenomenon known as Critical Slowing Down’ (CSD) to forecast imminent regime shifts in a system’s dynamics. CSD implies that systems approaching a tipping point need increasingly more time to recover from perturbations because of experiencing a loss of resilience. Their recovery to the stable state before the perturbation slows down [37]. CSD shows in statistical properties of the time series from a system’s dynamics taken at various states of equilibrium. An increase in autocorrelation at lag 1 (AC-1) for example, indicates a higher “short-term memory” of the system, related to changes in the correlation structure close to a tipping point [38]. Also, standard deviation (STD) and its trend-independent pendant, the coefficient of variation (CoV), tend to increase before a transition, caused by the systems inclination to deviate further from its stable state when losing stability. Additionally, the proximity to an alternative equilibrium and the resulting attraction can cause so called flickering, that is, asymmetries in variance and the occurrence of short jumps to states further away from the stable state and back. These effects show in changes in skewness (S) and kurtosis (K) of the analysed time series. Correlations over timescales longer than detectable with AC-1 can be measured by de-trended fluctuation analysis (DFA) [7]. And finally, serial correlations between successive time samples can also induce changes in spectral density, so called reddening (R), measured by spectral analysis (Formulas and references for these metrics are given in appendix A).

**Fig. 7:**
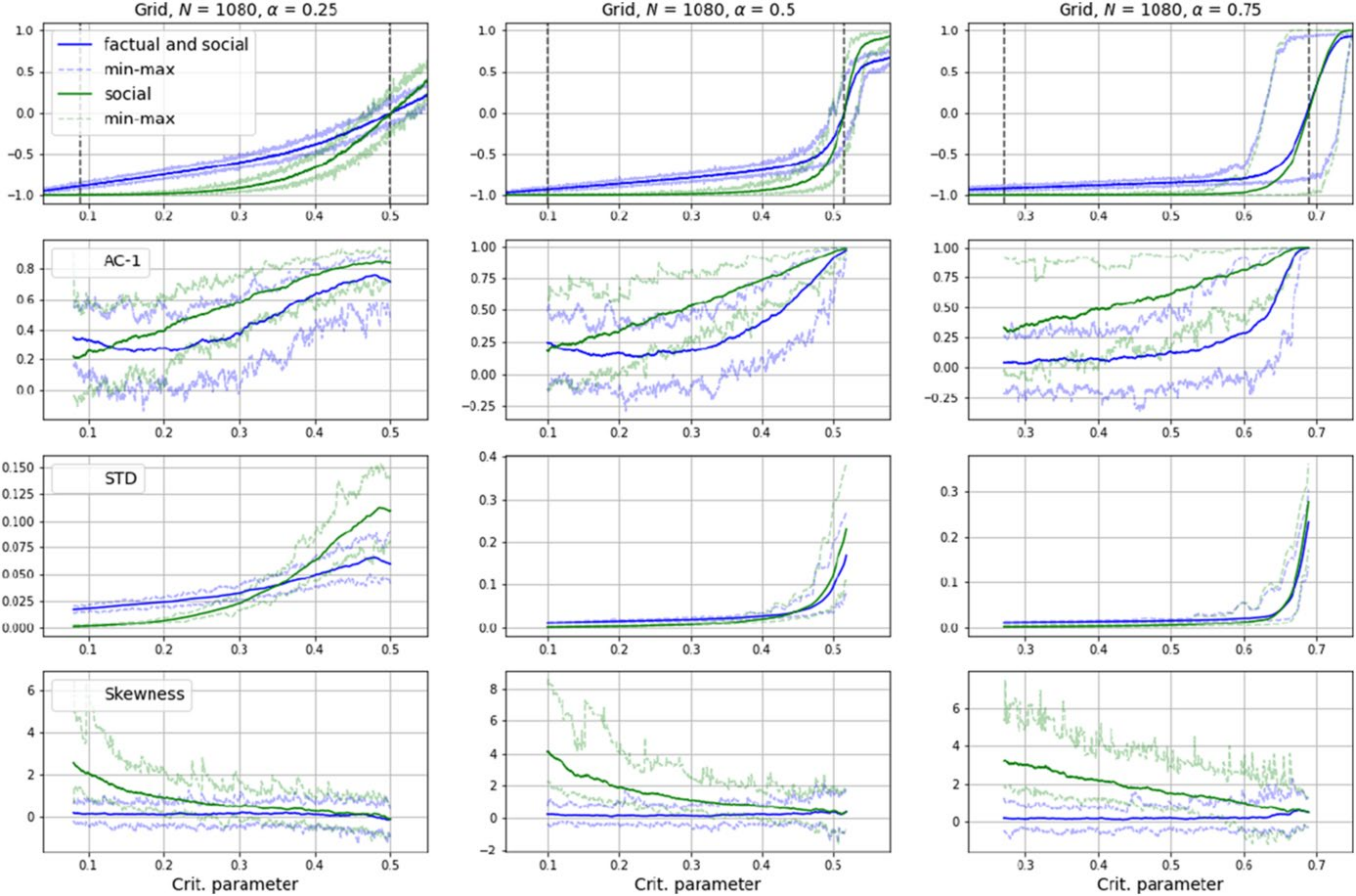
Results of EWS-analysis of time series generated with different $\alpha$-values determining the intensity of social influence. Signals from social influence on its own (green curves) appear to resonate more strongly than the aggregation of social and factual influence (blue curves). Autocorrelation at lag 1 (AC-1), Standard Deviation (STD) and Skewness (S). Results for Kurtosis, Coefficient of Variance, Detrended Fluctuation Analysis and Spectral Reddeninig are shown in appendix B

Figure 7 shows the results of an application of these metrics to time series generated with the model as described in “A model suggestion”, with different $\alpha$-values and a grid-positioned particle population of $P = 1080$ with periodic boundary conditions interacting in a Moore-neighbourhood (with equation-4-parameters: $a = 2$, $b = 1$). Linearly increasing the critical parameter from 0 to 1 makes the population shift spins in a rapid transition, the onset and steepness of which depends on the intensity of social influence (top row of Fig. 7). Both observables were applied to EWS-analysis on a window of 100 time steps being rolled over a before-transition part of 100 time series (indicated with dashed vertical lines in Fig. 7 top row), which in order to align the variance of tipping was taken 1500 steps backwards from where the transition reached a defined threshold value. The below-top-row plots in Fig. 7 show the averages of the metrics at the end of the rolling window. As can be seen, signals from social influence on its own (green curves) appear to resonate more strongly in nearly all EWS-metrics. What is more, the signals also become increasingly more discernible the higher the $\alpha$-value, that is, the higher the predominance of social influence. One may conclude hence that, if the setting allows it, it could make sense to focus EWS-analysis on signals from a system’s interaction dynamics. With regard to modelling, this implies that critical transition-prone systems should rather be modelled with agent-based than with equation-based methods.

We are aware though, that in most real-world cases the second observable, i.e. the intensity of social influence, will hardly ever be directly accessible. While a change of opinions for instance, as expressed in news reports or Twitter tweets might be detectable e.g. with automated text mining, the actual effect of corresponding social influence on these opinions will hardly ever be open to an observation with an appropriate signal-to-noise ratio. One might ask therefore, what, with regard to empirical studies, is to be gained with such differentiation of factual and social influences.

We propose that the actual benefit of this differentiation is in focusing analytic attention on particularities of social influence in critical transitions. As said, simulations in this respect, and in particular investigations on predicting transitions with EWS, are most commonly performed with equation-based methods, where the role and the kind of social influence can be missed. Even with quasi-agent-based models like the Ising, social influence remains obfuscated since analytically difficult to track. With a clear separation as suggested in our model, the social impact on the phenomenon of hysteresis is hard to overlook. It therewith insinuates further investigations in this regard, which may open up new aspects for EWS-analysis.

## EWS-analysis on a networked version

In this line, we implemented the model as described above in various network settings and examined their potential for EWS analysis. On the assumption that obtaining pure social influence data (as done in “EWS-analysis on the grid version”) will hardly be feasible empirically, we focused this network investigation on topological aspects instead. For this, we differentiated the particle population in the model with regard to the particles’ centrality in the generated networks, assuming that, at least in some settings, information about whether particles or agents are central in an interaction network may be empirically accessible.

**Fig. 8:**
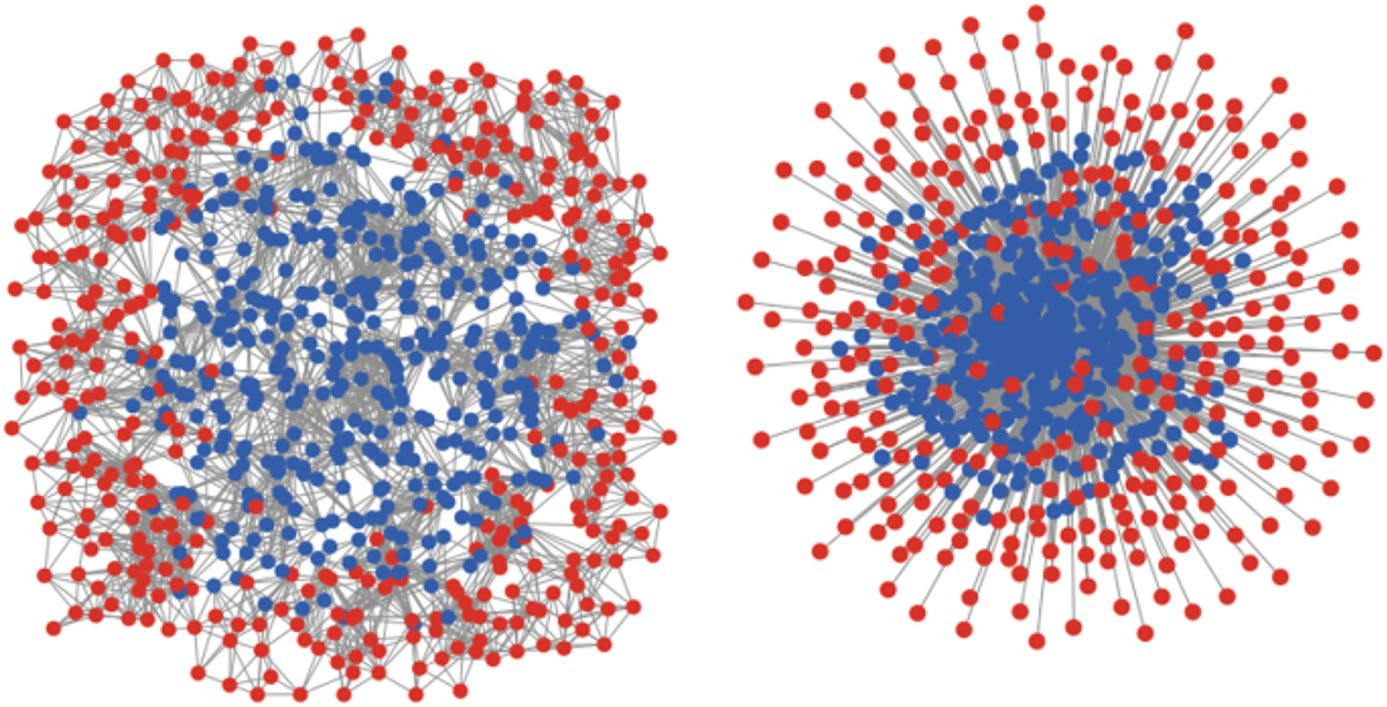
Left: average-degree network with degree = $10$; right: Goh-scale-free-network with $m=5$ and $\gamma =2$ generating an average degree of $10$, both Closeness-differentiated with nodes with above-median centrality in blue and nodes below-median in red

**Fig. 9:**
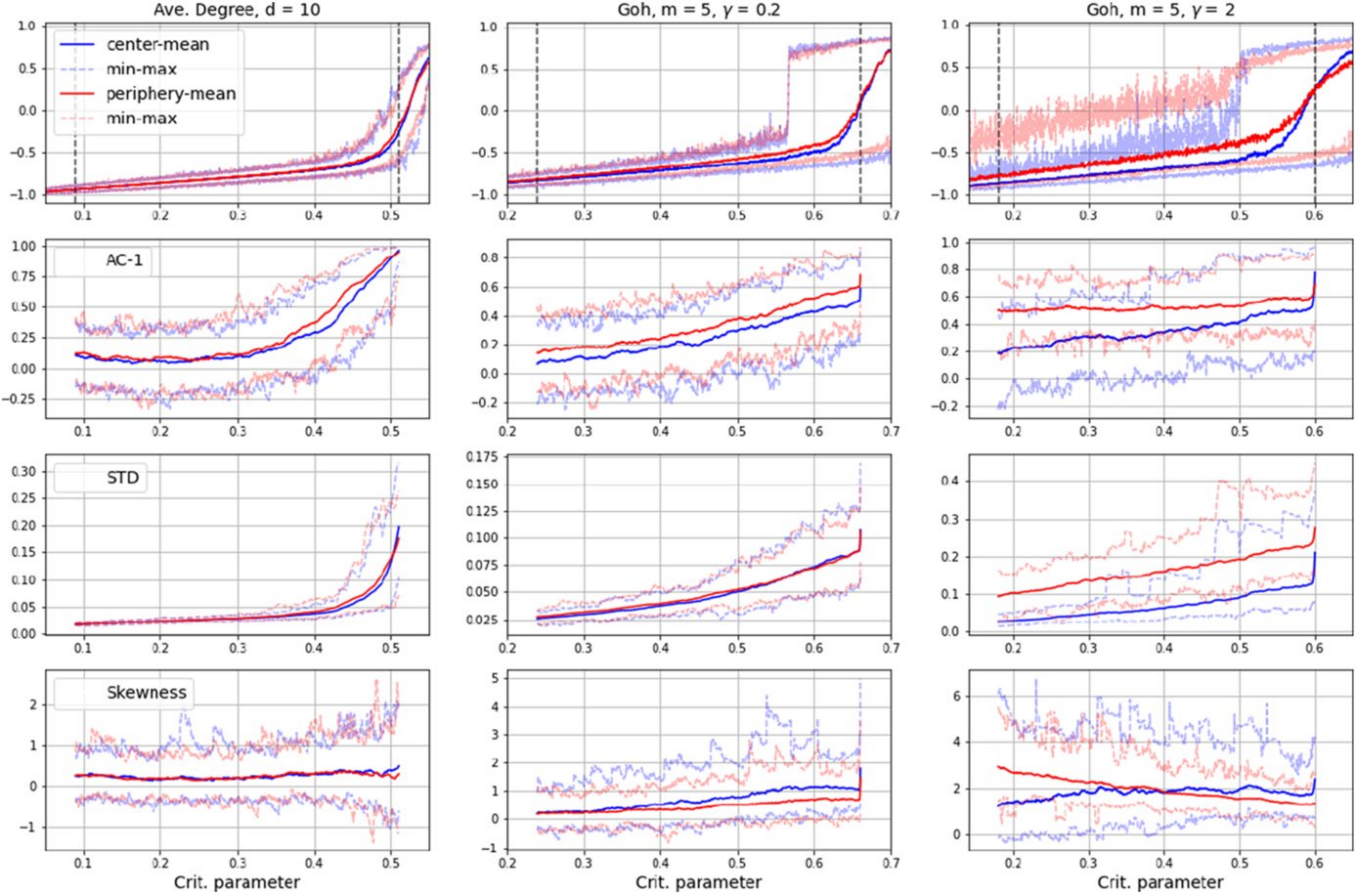
Results of EWS-analysis on time series from networked versions of the model as described in “A model suggestion”, differentiated in regard to the median of closeness-centrality into center (blue curves) and periphery (red curves), with EWS-metrics showing clearer signals in the onset of the transition when applied to the spin-average of peripheral nodes (red curves). Autocorrelation at lag 1 (AC-1), Standard Deviation (STD) and Skewness (S). Results for Kurtosis, Coefficient of Variance, Detrended Fluctuation Analysis and Spectral Reddeninig are shown in appendix B

We considered two different network topologies, the one being an average-degree network, with nodes having a uniformly distributed number of links to other nodes. And the other is a scale-free network implemented after the suggestion of Goh et al. [39], which, dependent on a parameter $\gamma$ varying between 0 and 2 generates networks with a distribution of link degrees obeying a power law $P(k)\approx k^{-\gamma }$ of which the (half of the) average link degree can be determined with a parameter $m$ using the algorithm explained in [40]. In both topologies, the population of $P=1000$ particles were differentiated with regard to centrality, taking the fraction with above-median centrality as “central” nodes (blue in Fig. 8) and the fraction with below median-centrality as “peripheral” nodes (red in Fig. 8). In Fig. 9, we show results of an EWS-analysis considering Closeness-centrality, which indicates the property of being “close” in terms of shortest paths to other nodes in a network [41]. Analogous tests for other centrality-metrics provided very similar results. The left image in Fig. 8 shows an instance of an average-degree network with degree = 10. The right image shows a Goh-scale-free-network with $\gamma =2$ and $m=5$ generating an average degree of 10, both Closeness-differentiated with nodes with above-median centrality in blue and nodes below-median in red.

On first view, the results of these EWS-analyses may seem counter-intuitive. Oriented on common diffusion theories [16], it should be assumed that the dynamics driving the system towards transition will gain momentum in the center of a network, where links are dense and numerous. The positive feedback of particles flipping faster the more particles already flipped, is thus expected to unfold from the central nodes in the network. In our experiments however, more distinct EWSs are to be gained from the periphery of the network, that is, from the less well-linked fraction of particles. What is more, the distinctness increases with the skewedness of a network’s link distribution. In the average-degree networks, where the link-disparity of central and peripheral nodes is rather small, the EWS-difference is noticeable but rather weak and could probably be overlooked (see left column rows for AC-1 or DFA in Fig. 9). In the scale-free network in contrast, where the link-distribution follows a power law (and can be adjusted with the $m$ and the $\gamma$-parameter), the effect shows quite clearly (middle and right columns in Fig. 9). With a highly skewed link-distribution (right column with $m=5$,$\gamma =2$) nearly all considered EWS-metrics gave significantly clearer signals in the onset of the transition when applied to the spin-average (i.e. factual plus social influence) of the peripheral nodes in the network (red curves) (see similar indications in [42].

As can be seen in the top row plots in Fig. 9 (particularly in the right one), the earlier start of spin agitation in the periphery (red) can be noticed on the time series themselves already. In particular the maximum values show clearly higher variance in the peripheral nodes (red). This allows for two interpretations. The fact that a less dense connectivity causes higher variance of spin states at lower values of the critical parameter, could imply an influence that works positively on spin agitation in this part of the network. But it may also imply the *absence* of a conservative force keeping particles from flipping, an influence that, due to low link density, gets less well propagated in this part of the network. We reason that this second influence is the *negative* feedback effect of a self-enforcing neighborhood inertness, which makes particles maintain their state and impedes spin shifts up to the point where larger neighborhood-clusters start changing their spins together. The propagation of this negative feedback is impeded in the sparsely linked network periphery, thus causing more resonance in those system dynamics that are measured with EWS. Other than the *positive* feedback of the “rich-get-richer”-dynamics, which are emphasized in social reinforcing or diffusion theories, the *negative* feedback is prone to be overseen in EBM-based representations of hysteretic critical transitions.

**Fig. 10:**
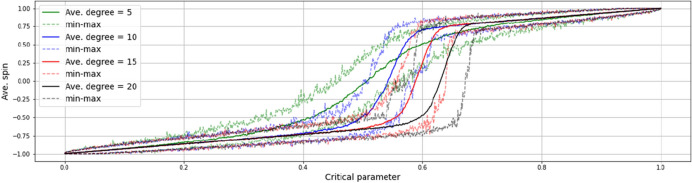
Comparison of transitions in different average degree networks (means of 20 instances)

At times, negative feedbacks seem to be outweighing the influence of positive feedbacks. A comparison of transitions in different average degree networks, depicted in Fig. 10, shows that the smaller the average degree, the flatter and more centered (i.e. less abrupt) the transition, but the higher the average degree, the more pronounced the hysteresis. Taking this together with the finding that more distinct EWS are to be gained from the periphery of a network, implies a weight of negative feedbacks in causing hysteretic transitions, which is heavier than usually mediated in diffusion theories.

## Generalizability check on networked Ising-model

An open question however, concerns the generalizability of this proposition, in particular with regard to the fact that our model foresees a strong non-linear interaction of particles, but is not necessarily governed by a combined saddle-node bifurcation, which often is taken as the theoretical cause of hysteresis when simulated with equation-based models (see e.g. equation 1). As mentioned, system states in the model at hand are taken instantly without equilibration, i.e. each spin is updated only once. The transitions it generates, thus may be fundamentally different to the ones of systems modeled with EBMs.

**Fig. 11:**
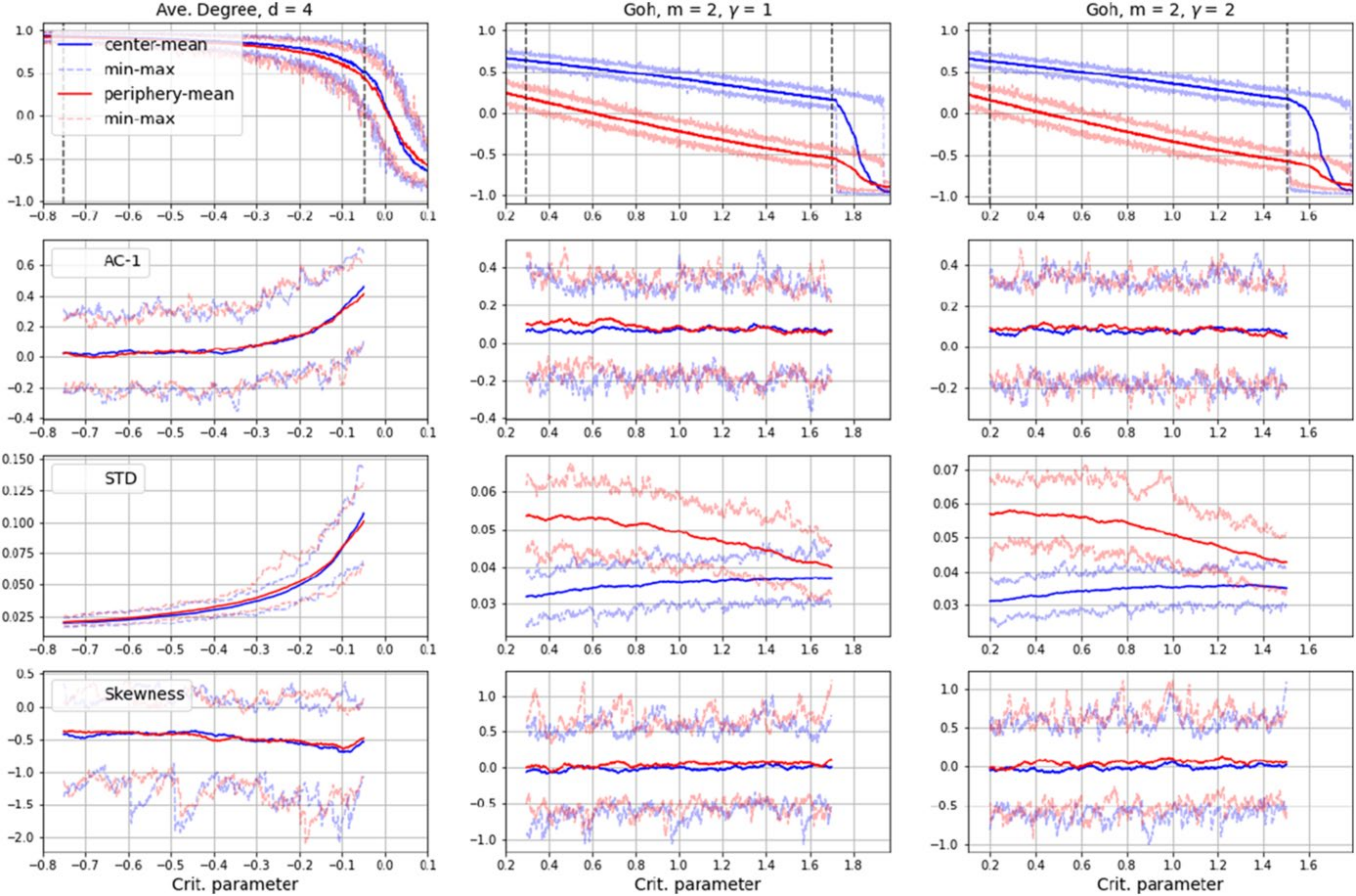
Results of EWS-analysis on time series from networked versions of the 2D-Ising model as analysed in “Critical transitions” in closeness-centrality differentiated network topologies as described in “EWS-analysis on the grid version”. Autocorrelation at lag 1 (AC-1), Standard Deviation (STD) and Skewness (S). Results for Kurtosis, Coefficient of Variance, Detrended Fluctuation Analysis and Spectral Reddeninig are shown in appendix B

In an attempt to assess generalizability, we implemented the 2D-Ising model as analysed in “Critical transitions” section in the same network topologies as described in "EWS-analysis on a networked version" section and applied EWS-analyses to analogue time series. Results are shown in Fig. 11. In this we encountered some subtleties. A problem arose from the fact that the original 2D-Ising is considered in a Von-Neumann neighborhood, which, when transferred to a randomly assigned link-structure, corresponds to a relatively sparsely linked network with some nodes having more than the average 4 link-neighbors, but others having less and some even having none at all. (To avoid a disintegrated network, nodes with no edges were randomly linked to one other node after generating the network, resulting in an average degree slightly higher than 4). While in the uniformly linked average degree network these differences in the link-structure have little effect and transitions occur more or less in similar parameter ranges as in the original Ising-model, the connection differences in the scale-free networks (indicated as ’Goh’ in Fig. 11) have more severe consequences. Here, the skewed degree distribution between center and periphery nodes causes a significant delay of the transitions, so that the actual system behavior does not correspond to the original Ising anymore (see top row in Fig. 11). What is more, the fact that spin orientations are subject to equilibration in the Ising-model and thus balancing the effects of neighborhood interaction each time before a consecutive parameter step takes effect causes rather large initial differences between center and periphery particles in magnetization (see middle and right top-row plots in Fig. 11). On the one hand, this could be interpreted as supporting our suggestion, namely that spin agitation takes off in the periphery, where conservative negative feedbacks are impeded due to low link density. Additionally, the initial difference in magnetization seems to cause a sort of convergence of EWS-metrics for central and peripheral nodes, as particularly visible in the plots for STD, CoV and Reddening, suggesting a significant difference between center and periphery in the onset of a transition. On the other hand, the majority of EWS-metrics does not show clear signals for indicating an imminent state change in this setting. In particular, AC-1, Skewness, Kurtosis and DFA remain rather static, although the actual time series clearly decline in the considered range (middle and right top-row plot in Fig. 11).

## Discussion

Our experiments with a simple, but highly flexible spin model used to generate time series of critical transitions analysed with EWS-methods, give reason to propose that:

- (a.) neighborhood, i.e. social interaction, and in particular negative, not positive feedback effects may be prime drivers in critical transitions; and
- (b.) peripheral particles in an interaction network could be particularly promising objects when looking for indications of an imminence of such transitions.

These propositions however, are based on a system that, while having strong analogies to systems commonly used to study saddle-node bifurcations, differs in the way convergence to equilibrium states of the system is generated. The fact that the critical parameter is used here as a probability rather than a threshold complicates a direct comparison of the results with those of other systems with hysteresis. Also, the question of how the number of neighbours considered in social interaction affects the delay of critical transitions, and to what extent thus a network implementation of the Ising model is physically justified, opens up fields for research and asks for further investigations. The attempt to test our propositions using a networked version of the Ising model may therefore be considered debatable. To some extent hence, the question about the generalizability of our findings has to be left open. Although some support for our proposition seems to be gained from the networked Ising-model, the EWS-signals appear not to be sufficiently explicit for a clear reconfirmation, implying the need for further research, possibly into alternative models commonly used for investigations on the predictability of critical transitions. Nevertheless, we do consider our results as a strong indication that critical transitions, and in particular such that are subject to hysteresis, are primarily driven by complex neighbourhood interactions, the details of which remain veiled when considered with equation-based methods. EWS-analysis therefore should consider agent-based methods as an alternative or at least complementary to equation-based models.

## Figures and Tables

**Fig. 12:**
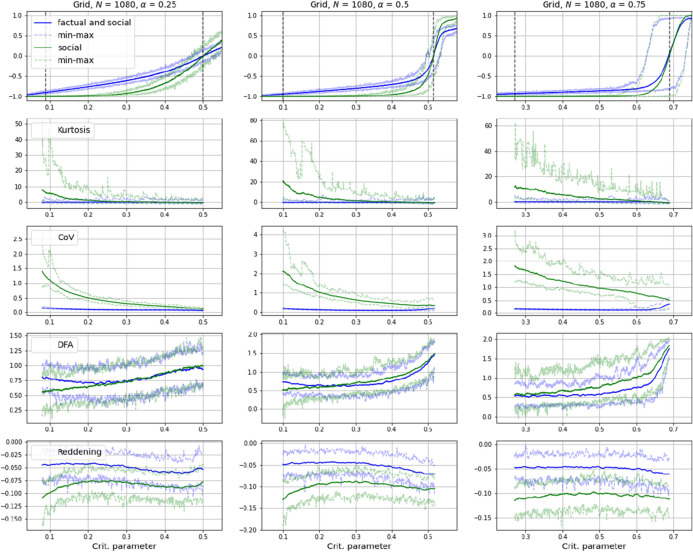
Results of EWS-analysis (Kurtosis, Coefficient of Variance, Detrended Fluctuation Analysis and Spectral Reddeninig) of time series generated with different $\alpha$-values determining the intensity of social influence. Supplementary to Fig. 7

**Fig. 13:**
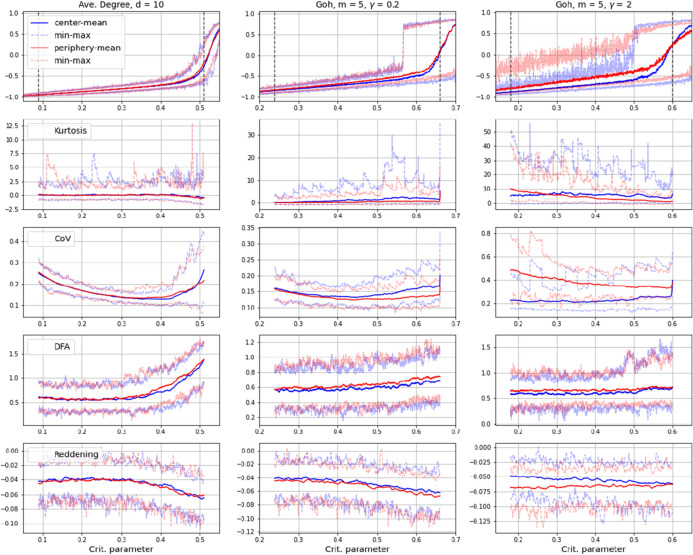
Results of EWS-analysis (Kurtosis, Coefficient of Variance, Detrended Fluctuation Analysis and Spectral Reddeninig) on time series from networked versions of the model as described in “A model suggestion”, differentiated in regard to the median of closeness-centrality into center (blue curves) and periphery (red curves). Supplementary to Fig. 9

**Fig. 14:**
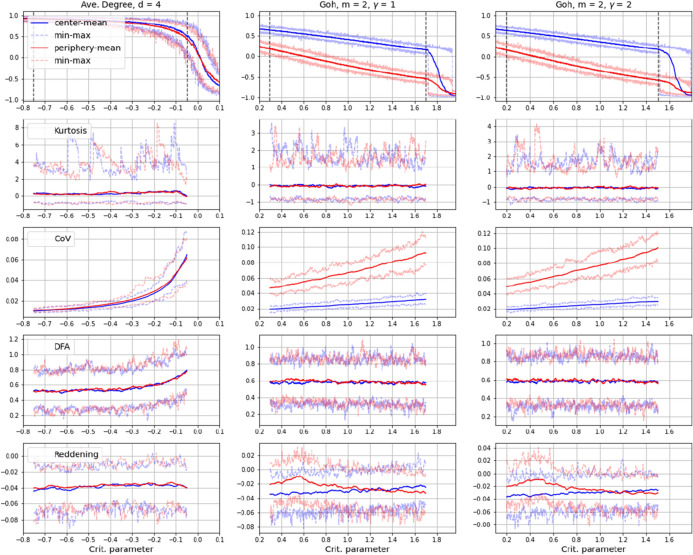
Results of EWS-analysis (Kurtosis, Coefficient of Variance, Detrended Fluctuation Analysis and Spectral Reddeninig) on time series from networked versions of the 2D-Ising model as analysed in “Critical transitions” in closeness-centrality differentiated network topologies as described in section 5. Supplementary to Fig. 11

## Appendix A

The standard EWS, Autocorrelation at lag 1 (AC-1), Standard deviation (STD), Skewness (S) and Kurtosis (K), as suggested among other in [37], are calculated for the time series $X$ containing $N$ elements labeled $x_i$ using the following equations:

Standard deviation

6 $$\begin{aligned} \sigma (X) =\sqrt{\frac{\sum _{i=1}^N(x_i - {\bar{x}})^2}{N - 1}} \end{aligned}$$

Autocorrelation at lag 1

7 $$\begin{aligned} AC1(X) =\frac{1}{N-1}\sum _{i=1}^N\left( \frac{x_i - {\bar{x}}}{\sigma _x}\right) \left( \frac{y_i - {\bar{y}}}{\sigma _y}\right) \end{aligned}$$

Skewness

8 $$\begin{aligned} S(X) =\frac{\frac{1}{N}\sum _{i=1}^N(x_i - {\bar{x}})^3}{\sigma _X^3} \end{aligned}$$

Kurtosis

9 $$\begin{aligned} K(X) =\frac{\frac{1}{N}\sum _{i=1}^N(x_i - {\bar{x}})^4}{\left( \frac{1}{N}\sum _{i=1}^N(x_i - {\bar{x}})^2\right) ^2}-3 \end{aligned}$$

Coefficient of Variation

10 $$\begin{aligned} CV(X) =\frac{\sigma (X)}{{\bar{X}}+1} \end{aligned}$$

Here ${\bar{x}}$ denotes the mean of $x$ and $y_i$ are the elements of a time series that is generated by shifting $X$ by 1, so that $y_i = x_{i-1}$

The application of detrended fluctuation analysis (DFA) and the analysis of spectral reddening (R) is explained in [7] and [40].

## Appendix B

See Figs. 12, 13, 14.

## Footnotes

1 $norm = \frac{x - minX}{maxX - minX}$, with $minX$ and $maxX$ being the extrems of $(\alpha f(s+nb) + (1-\alpha ) c)$,
